# The genomic landscape of Ménière's disease: a path to endolymphatic hydrops

**DOI:** 10.1186/s12864-024-10552-3

**Published:** 2024-06-28

**Authors:** Kathleen M. Fisch, Sara Brin Rosenthal, Adam Mark, Roman Sasik, Chanond A. Nasamran, Royce Clifford, M. Jennifer Derebery, Ely Boussaty, Kristen Jepsen, Jeffrey Harris, Rick A. Friedman

**Affiliations:** 1 Center for Computational Biology & Bioinformatics, University of California, San Diego, La Jolla, CA USA; 2 Department of Obstetrics, Gynecology & Reproductive Sciences, University of California, San Diego, La Jolla, CA USA; 3 Department of Otolaryngology, Head & Neck Surgery, University of California, San Diego, La Jolla, CA USA; 4 Research Department, VA Hospitals, San Diego, CA USA; 5 House Institute Foundation, Los Angeles, CA USA; 6 Institute for Genomic Medicine, University of California, San Diego, La Jolla, CA USA

**Keywords:** Ménière's disease, Whole genome sequencing, Systems biology, Network analysis, Gene discovery

## Abstract

**Background:**

Ménière's disease (MD) is a disorder of the inner ear that causes episodic bouts of severe dizziness, roaring tinnitus, and fluctuating hearing loss. To date, no targeted therapy exists. As such, we have undertaken a large whole genome sequencing study on carefully phenotyped unilateral MD patients with the goal of gene/pathway discovery and a move towards targeted intervention. This study was a retrospective review of patients with a history of Ménière's disease. Genomic DNA, acquired from saliva samples, was purified and subjected to whole genome sequencing.

**Results:**

Stringent variant calling, performed on 511 samples passing quality checks, followed by gene-based filtering by recurrence and proximity in molecular interaction networks, led to 481 high priority MD genes. These high priority genes, including *MPHOSPH8, MYO18A, TRIOBP, OTOGL, TNC,* and *MYO6,* were previously implicated in hearing loss, balance, and cochlear function, and were significantly enriched in common variant studies of hearing loss. Validation in an independent MD cohort confirmed 82 recurrent genes. Pathway analysis pointed to cell–cell adhesion, extracellular matrix, and cellular energy maintenance as key mediators of MD. Furthermore, the MD-prioritized genes were highly expressed in human inner ear hair cells and dark/vestibular cells, and were differentially expressed in a mouse model of hearing loss.

**Conclusion:**

By enabling the development of model systems that may lead to targeted therapies and MD screening panels, the genes and variants identified in this study will inform diagnosis and treatment of MD.

**Supplementary Information:**

The online version contains supplementary material available at 10.1186/s12864-024-10552-3.

## Background

Ménière's disease (MD), first described by Prosper Ménière in 1861 [1], is a disorder of the inner ear that causes intermittent bouts of severe dizziness, roaring tinnitus, and fluctuating hearing loss. The disease prevalence ranges between 3.5 per 100,000 and 513 per 100,000, has a female to male ratio of 1.89 to 1 [2], and is most often sporadic but can occur in a familial form in roughly 5% of cases [3]. Although the cause is unknown, human temporal bone studies have linked MD symptoms to elevated pressure within the inner ear—specifically, the endolymphatic cochlear compartment (scala media) and endolymphatic duct. It is believed that this endolymphatic hydrops begins with derangement of the ionic composition of the scala media. The symptoms of the disease—tinnitus, vertigo, and hearing loss—are managed with salt restriction, diuretics, vestibular suppressants, and corticosteroids and possible surgical intervention in incapacitating cases. Nonetheless, 60 percent of patients progress to severe hearing loss and persistent disequilibrium. To date, the true etiology of the disease remains unknown, and no targeted therapy exists.

The National Institute on Deafness and Other Communication Disorders (NIDCD) estimates that there are 615,000 Americans with MD and the disease accounts for 45,500 patient visits each year. Although very little literature exists on the socioeconomic impact of MD, a study from Sweden followed 19 patients over a 3-year period to assess the impact on productivity [4]. It was concluded that the costs to society and the patients were substantial, with 1,536 days of sick leave requested by these 19 subjects. In addition to these lost days, there is the tremendous cost of surgery, lost productivity due to agoraphobia and the impact of drop attacks on vocation, driving, and the activities of daily living [5]. Another study found MD to be one of the most debilitating diseases experienced by people who survive any illness [6]. Taken together, these data suggest that patients with this disease are in dire need of therapeutics.

The genetic etiology of MD is supported by a prevalence of familial cases [7–10], an over-representation of MD in people of Caucasian ancestry [11], and candidate gene studies [12–14]. We previously published a genome-wide analysis of patients with MD disease and demonstrated a clear ancestral predilection (Caucasians) supporting the notion of a genetic etiology [11]. As a result, we have undertaken the largest whole genome sequencing study to date on carefully diagnosed unilateral MD patients with the goal of gene/pathway discovery and a move towards targeted interventions for this disorder.

In this manuscript we present the first whole genome sequence analysis for rare damaging genetic variants associated with well-characterized classical MD consisting of attacks of fluctuating unilateral low frequency hearing loss, roaring tinnitus, and vertigo. Analysis of rare damaging variants in this cohort reveals 481 high priority MD genes, in which we find many prior associations with hearing loss, balance, and cochlear function.

## Results

### Recurrent rare variants observed in Ménière's disease

**Fig. 1:**
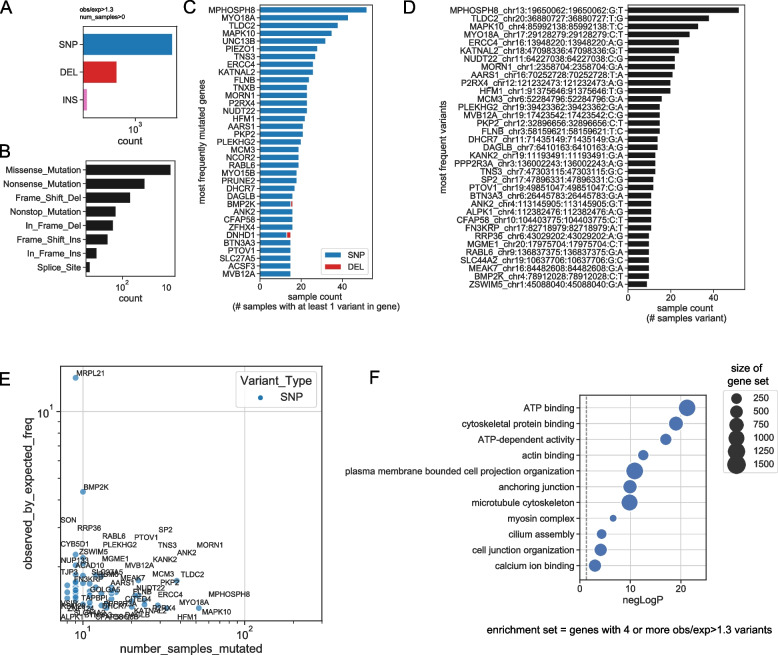
Summary of variants in study population. **A** Bar chart displaying variants by type, after application of all filters. **B** Bar chart displaying variants by function. **C** Bar chart displaying 35 most frequently mutated genes. **D** Bar chart displaying the 35 most recurrent variants. **E** Scatterplot showing the top 50 most frequently mutated genes, with the number of impacted samples on the x-axis, and the ratio of observed frequency to expected frequency on the y-axis. **F** Scatterplot showing select enriched GO terms in the set of genes with > = 4 variants with obs/exp > 1.3 per gene, ranked by -log(p) (hypergeometric test)

In 511 MD individuals, we observed 16,790 distinct rare damaging missense and loss of function (LOF) variants (Table S1). Of these variants, 11,209 (66.8%) were observed at a frequency in the study population more than 1.3-fold higher than the expected rate in the general population as observed in gnomad or were novel variants. These unusually frequent variants (UFVs) formed the basis of our analysis. Single nucleotide polymorphisms (SNPs) constituted 97.7 of these UFVs (10,945), with a small number of deletions (234) and insertions (30) (Fig. 1A). Most variants were missense (10,132), with a smaller number of nonsense variants (775), frame-shift deletions (199), nonstop (41), in-frame deletions (31), frame-shift insertions (22), in-frame insertions (6), and splice site variants (3) (Fig. 1A). While ancestry information was not available for the study cohort, we were able to infer ancestry by aligning with 1000 Genomes [15] (Figures S4A). We did not observe ancestry-specific differences in number of variants per sample after filtering, so we retained all samples in the analysis (Figures S4B-C).

The most recurrent UFV was a missense mutation in the *MPHOSPH8* gene (rs75390100), with 52 occurrences in the study population, corresponding to a study allele frequency of (5.1%) (Fig. 1D). The study frequency is 1.3 fold increased over expectation (3.9% in gnomad database). While this gene has not previously been characterized in human hearing loss, heterozygous alteration of *MPHOSPH8* in mouse results in abnormal auditory brainstem response[16]. *MYO18A* had many distinct UFVs, with 9 separate UFVs observed, comprising a total of 42 samples (Fig. 1C). The top 50 genes ranked by total number of variants displayed a range of observed/expected frequency (Fig. 1E). We note that these variants may represent an increased predisposition for the disease, but are alone not specific enough for diagnosis. We defined a highly recurrent gene set, comprising 1098 genes with 4 or more UFVs. These highly recurrent genes were significantly enriched in many gene ontology pathways relevant to inner ear function, including ATP binding [17], actin binding [18], cilium assembly, myosin pathways, cytoskeleton organization, cell junctions [19], and calcium signaling [20] (Fig. 1F; Table S2).

### Prioritization of mutated genes with network analysis identifies genes and pathways consistent with the MD phenotype

**Fig. 2:**
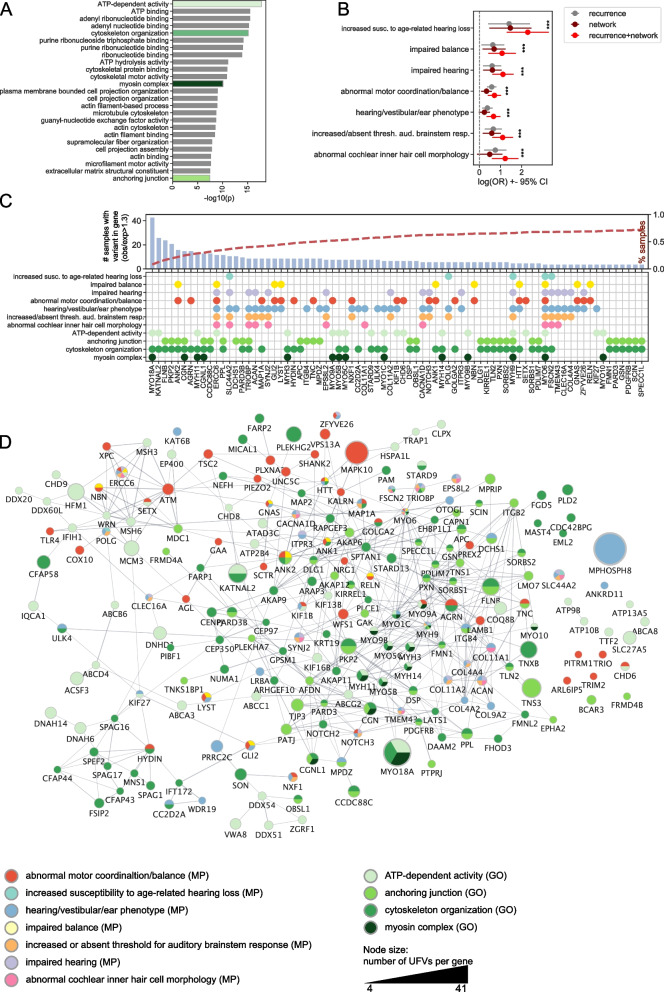
Network prioritization of candidate genes. **A** Barplot showing top GO pathways for recurrence + network gene set. **B** Scatterplot showing the log odds ratio of enrichment between relevant terms in the mammalian phenotype ontology and MD genes filtered in one of three ways: 1) Recurrence = gene has > = 4 unexpected variants (unexpected = obs/exp > 1.3); 2) Network = netprop z > 3; 3) Recurrence + netprop = gene has > = 4 unexpected variants & netprop z > 3 (N = 481 genes). **C** Barplot showing the highest frequency genes meeting filtering criteria which appear in 2 or more relevant pathways/phenotypes. Right axis (red dotted line) shows cumulative sum of % samples explained by variants in genes. **D** Subset of the recurrence + netprop set of genes from selected terms and pathways most relevant to MD. Node color indicates the pathway(s) membership. Node size indicates the number of unexpected variants per gene. Medium confidence STRING edges are shown. https://www.ndexbio.org/viewer/networks/c6b7c224-41ed-11ee-aa50-005056ae23aa

In lieu of traditional gene burden testing [21], which was not possible here because a control population was not available, we aggregated gene-level scores based on network-propagation [22] and recurrence of UFVs. Network propagation serves as an amplifier of genetic associations, by highlighting groups of genes from the input set which have more connections than expected by chance, and thus likely represent a biological pathway which plays a role in the disease at hand. Genes which have many variants, but are not highly connected to other genes with variants, and may be false positives, are down weighted. When the network propagation scores were integrated with recurrence scores (network z > 3 and recurrence > = 4, *N* = 481 genes), the significantly enriched pathways were similar to those identified from recurrence alone, including ATP binding, myosin complex, and cytoskeleton organization (Fig. 2A; Table S2).

To evaluate success at boosting the signal in the data with network propagation, we turned to a public database which connects mouse genotypes to resulting phenotypes[23]. Genes associated with relevant phenotypes (related to hearing, balance, or auditory processes) were evaluated for significant overlap with genes in our data. We tested 3 filtering criteria: network alone (network z > 3; *N* = 1,073 genes), recurrence alone (recurrence > = 4, *N* = 1098 genes), and network + recurrence (network z > 3 and recurrence > = 4, *N* = 481 genes). We found that all 3 filtering criteria resulted in significant enrichment for cochleovestibular phenotypes (Fig. 2B; *p* < 0.05). Increased susceptibility to age-related hearing loss (*p* = 7E-6), impaired balance (*p* = 1E-3), impaired hearing (*p* = 2E-5), abnormal motor coordination/balance (2E-5), and abnormal cochlear inner hair cell morphology (*p* = 2E-4) were particularly highly enriched (Fig. 2B,C; Table S3, Table S4). In general, the network + recurrence gene set performed best (Fig. 2B), suggesting that the convergence of high recurrence and network information yields the highest ratio of signal to noise. The enrichment results were not sensitive to choice of threshold (Figure S2). This network + recurrence gene set was used for further analysis.

To build an MD-prioritized gene network, we intersected the network + recurrence prioritized set with genes found in relevant pathways and phenotypes (Fig. 2C,D). Some genes (*MYO6*, *MYH9, ERCC6*) were identified in nearly every relevant phenotype and/or pathway, while others (*OTOGL, TRIOBP, COL11A2, COL4A3)*, have well established connections to hearing disorders in the literature[24–27], yet they were not the most recurrently mutated genes, with 12 or fewer samples having a qualifying variant. We suspect that these genes may be less tolerant to variation, or that variants in these genes more commonly result in other hearing disorders. Highly recurrent genes, such as *MYO18A*, and *KATNAL2*, impact both ATP-dependent activity, and cytoskeleton organization, but are not well characterized in relation to hearing and balance disorders, and may represent novel MD genes.

Many genes in the myosin family were impacted in the network + recurrence gene set(*MYO9B, MYO5C, MYO5B, MYH3, MYH14, MYH11,* etc.; Fig. 2C,D). Genes in the myosin family have well documented relationships to hearing impairment, as they are instrumental in development and maintenance of auditory hair cell stereocilia [28, 29]. Variants in *MYO18A* had particularly high recurrence in the study cohort, with 29 occurrences of the missense mutation rs117024203, an additional 7 observations of the missense mutation rs76590796, 4 other rarer missense mutations observed a single time, and one nonsense mutation. Variants in MYO18A have previously been implicated in a study of Swedish MD and tinnitus extreme phenotypes [30].

### Validation of high priority variants and genes in independent replication cohorts

**Fig. 3:**
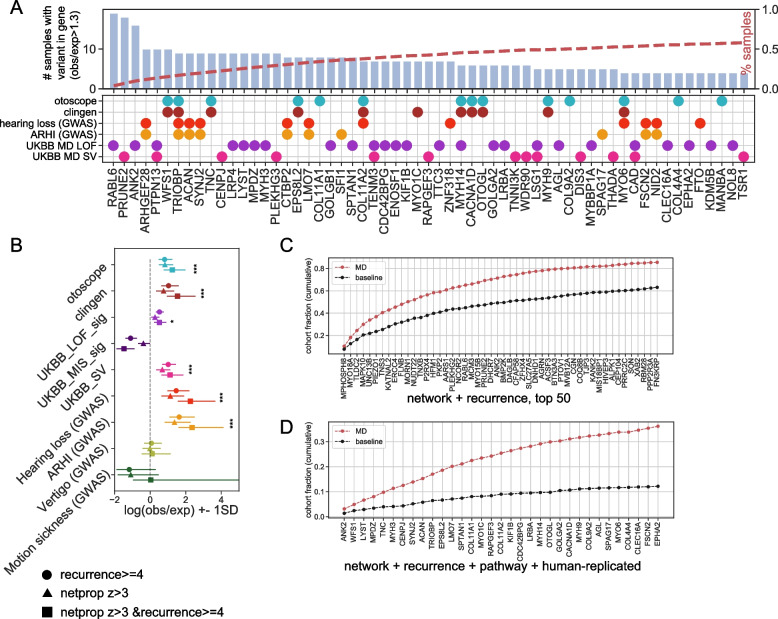
Diagnostic value and validation. **A** Bar chart showing top replicated genes from external human databases. Right axis (red dotted line) shows cumulative sum of % samples explained by variants in genes. Genes ranked by recurrence. Scatterplot below shows gene set membership. **B** Enrichment with external human databases: log(obs/exp) + -1SD. Hypergeometric test for enrichment with gene sets shown. *p < 0.05, **p < 0.01, ***p < 0.005. **C** Cumulative percentage of MD samples explained by UFVs in top 50 recurrent + network (red), ranked by recurrence, compared to the baseline expectation given allele frequencies in general population (black; gnomad). 50% of MD cohort explained by 11 genes. We would expect to recover 33% of a control cohort with these same variants. **D** Cumulative percentage of MD samples explained by UFVs in high priority genes from pathway and human replication analysis (red), compared to the baseline expectation given allele frequencies in general population (black; gnomad). 36% of MD cohort explained by 33 high priority genes. We would expect to recover 12% of a control cohort with these same variants

We cross-referenced the network + recurrence prioritized genes for enrichment in publicly available human datasets. These include: 1. The OtoSCOPE v9 gene panel, a diagnostic tool to evaluate presence of variants in genes involved in non-syndromic and select types of syndromic hearing loss [31] (otoscope); 2. Clinically curated pathogenic variants in 142 human genes related to hearing loss [32] (Clingen); 3. Variants identified from UKBB MD (self-reported, exomes), from gene burden tests on LOF variants (UKBB_LOF_sig), missense variants (UKBB_MIS_sig), or from single-variant gene tests (UKBB_SV); and 4. Genes associated with variants identified from human GWAS on relevant phenotypes (hearing loss, age-related hearing impairment, vertigo, and motion sickness) from the GWAS catalog. Of these gene sets, the network + recurrence gene sets were significantly enriched for genes in the otoscope panel, ClinGen, the UKBB_LOF_MD, UKBB MD single-variant analysis, GWAS hearing loss, and GWAS age-related hearing impairment. UKBB_MIS_MD, GWAS vertigo, and GWAS motion sickness were not significantly enriched. Similar to the results from the mouse variant database, the network + recurrence gene set demonstrated the best performance in recovery of human-relevant gene sets (Fig. 3A-B; Table S4).

*RABL6*, *ANK2*, and *MYH3* were highly recurrent, and only found in the UKBB MD LOF set (Fig. 3A). These genes may be specific/unique to MD, as compared to the more general ‘hearing loss’ phenotype. A total of 82 recurrent genes were significant in the UKBB MD LOF and/or SV set (Figure S4), representing genes most likely to be MD-specific. The genes *ARHGEF28*, *SYNJ2*, and *ACAN* have been previously implicated in common variant studies of hearing loss (GWAS). *TRIOBP*, *MYO6*, and *COL11A2* were found in both clinical gene sets (otoscope and clingen), and common variant studies of hearing loss (GWAS) (Fig. 3A-B). *TNC*, and *WFS1*, were highly recurrent, and found only in the otoscope and clingen gene sets. Taken together, these results illustrate the multifaceted and complex nature of MD; far from being a monogenic disease, MD may manifest from variation in many different genes and/or pathways.

### Predictive utility of MD-prioritized genes

Along with pointing to high priority therapeutic targets, the genes identified in this study represent a path to improved diagnosis. 50% of the study cohort have at least one qualifying variant in the top 11 out of 481 genes ranked by network + recurrence (Fig. 3C). However, we would expect 33% of a control population to be identified as false positives using these same variants, assuming allele frequencies from the general population (gnomad). We note that this estimate of false positive rate is likely an underestimate, since the variants selected for inclusion were chosen in part due to their high ratio of observed to expected allele frequency. In order to reduce the false positive rate, we applied stricter inclusion criteria, by restricting the 481 genes to a subset of those that resulted in 33 genes that had roles in relevant pathways and were replicated in human gene sets (33 genes out of the 481 genes ranked by network + recurrence). Here the fraction of study cohort recovered decreased to 36%, but the expected false positive rate is reduced to 12%, a marked decrease (Fig. 3D). In addition to genes discussed previously (*ANK2, WFS1, TNC, TRIOBP*, and *OTOGL*), this group includes *LRBA*, a gene required for maintenance of cochlear hair cells [33], and *MPDZ*, a gene implicated in autosomal recessive nonsyndromic hearing impairment [34] This group of genes, with more conservative selection criteria, may form the basis of a new screening panel for MD.

### Expression of MD-prioritized genes in hearing-impaired mouse inner ear cell types

**Fig. 4:**
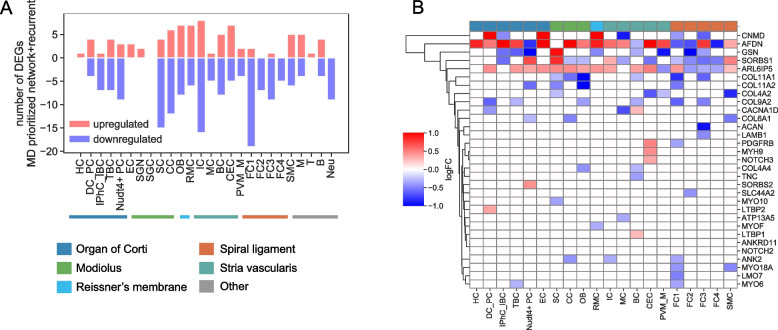
Replication in model of hearing impaired mice **A**) Number of MD-prioritized genes which are upregulated (red) and downregulated (blue) in ears of hearing impaired mice compared to healthy controls. Data are shown by otic cell types. **B** Select significantly differentially expressed genes per otic cell type are shown in the heatmap, with red indicating upregulation in the hearing-impaired mice, and blue indicating downregulation in the hearing-impaired mice. HC: Hair Cell, DC_PC: Deiter cell and pillar cell, IPhC_IBC: Inner phalangeal cell/Inner border cell, TBC: Tympanic border cell, Nudt4 + _PC: Nudt4 + pillar cell, EC: Epithelial cell, SGN: Spiral ganglion neuron, SGC: Satellite glial cell, SC: Schwann cell, CC: Chondrocyte, OB: Osteoblast, RMC: Reissner’s membrane cell, PVM_M: Perivascular resident macrophage-like melanocyte, FC1: Fibrocyte1, FC2: Fibrocyte2, FC3: Fibrocyte3, FC4: Fibrocyte4, SMC: Smooth muscle cell, M: Macrophage, T: T cell, B: B cell, Neu: Granulocytes/neutrophils

To probe the functional relevance of genes prioritized from the network and recurrence analysis, we turned to a model of age-related hearing impairment in mice [35], since an analogous dataset does not exist for humans. MD-prioritized genes (481 network + recurrent set) were significantly differentially expressed in select cell types in hearing impaired mice relative to healthy controls (Fig. 4A,B). Specifically, the most dysregulation was observed in celltypes within the modiolus, as well as a subtype of the spiral ligament (Fig. 4A). *MYO18A* was significantly downregulated in hearing impaired mice in fibrocytes and smooth muscle cells in the spiral ligament, an area associated with mediation of cochlear ion homeostasis. Genes in the collagen family were also significantly downregulated in these cell types. These include *COL11A1, COL11A2, COL4A2, COL9A2*, and *COL6A1*, in which rare recurrent variants are seen in 30 MD patients in the study cohort. *SORBS1* and *SORBS2*, genes which are known to play a role in formation of actin stress fibers and cytoskeleton organization, are significantly upregulated in hearing impaired mice in a subtype of the organ of Corti. 11 rare and recurrent variants were identified in *SORBS1* and *SORBS2* in the study cohort, and *SORBS1* was also significantly associated with severe tinnitus in a recent study[30]. The dysregulation of MD genes in the model of hearing impairment in mice provides additional support for the functional relevance of these genes, and suggests expression in specific inner ear cell types and relevant areas of the cochlea to MD.

### Cell-type specificity of MD-prioritized genes in human inner ear cell types

**Fig. 5:**
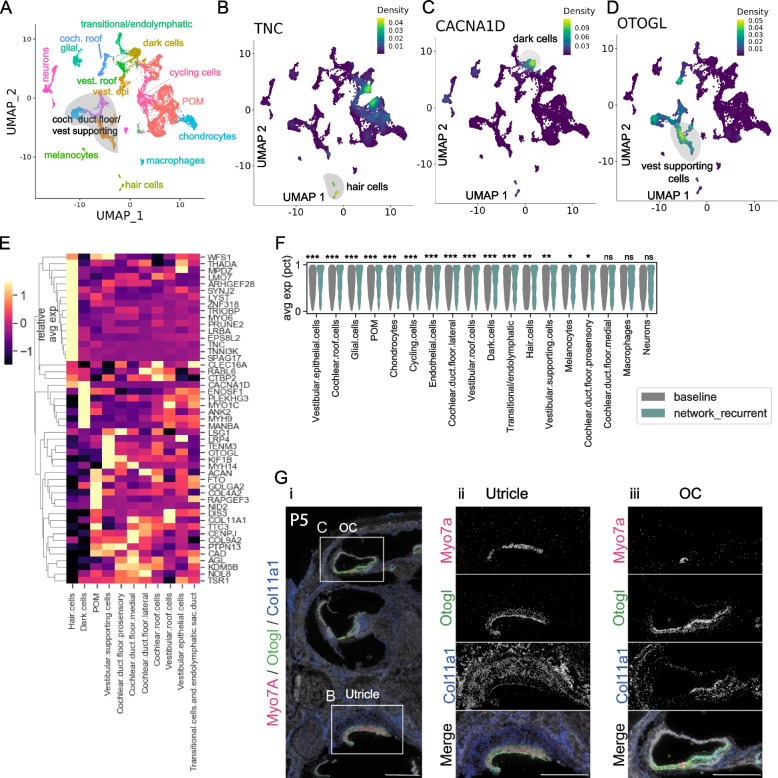
Cell-type specific expression of MD genes. **A**-**D** UMAP cell types and expression levels of select genes from the human inner ear atlas[36]. POM: periotic mesenchyme. **E** Relative average expression in human inner ear atlas cell types for select MD prioritized genes which validated in human databases (genes from Fig. 3A). **F** Violinplots showing the average percentile expression, per human inner ear atlas celltype, for genes which have any variant in the study cohort (baseline; gray), and for genes in the network + recurrent prioritized set (network_recurrent; green). **G** MERFISH spatial expression of two genes of interest; Otogl, Col11a1, along with Myo7a to indicate hair cells. Panels show i) low magnification view of the cochlea, and zoomed in expression of genes of interest in the utricle (ii) and organ of Corti at the mid-apical turn(OC) (iii). Scale bar: 250 µm. *** FDR < 0.001, ** FDR < 0.01, * FDR < 0.05, ns not significant; wilcoxon rank sum test, benjamini hochberg correction for multiple tests

In a single cell atlas of human inner ear cell types [36], some MD prioritized genes demonstrate high cell-type specificity (Fig. 5A-E). *TNC* is highly expressed in hair cells, while *CACNA1D* is highly specific to dark cells, and *OTOGL* is localized to vestibular supporting cells. In particular, many of the human-validated genes were highly specific to hair cells (including TRIOBP, MYO6, PRUNE2, LMO7) (Fig. 5E). Other genes were highly specific to dark cells, and various vestibular/supporting cells (CACNA1D, MYH9, OTOGL, ANK2) (Fig. 5E). Dark cells are epithelial cells which line the endolymphatic space and utricle, and are involved in the production of endolymph. As endolymphatic hydrops is thought to be a major component of MD, genes which are mutated in MD patients, and which are expressed highly in dark cells or other endolymphatic and vestibular supporting cells are of particular interest. MERFISH spatial expression of OTOGL confirms localization to the utricle and organ of Corti (Fig. 5G), consistent with findings of Otogl and Otog expression in the mouse cochlea using RNAscope from Jean et al. 2023 [37]. These data suggest localization to cell types in which the effect of the MD genes and variants may have the highest impact.

On the other hand, compared to a baseline of all genes in the study cohort with at least one variant, MD network + recurrent prioritized genes were significantly more highly expressed in most cell types (Fig. 5F; rank-sum test, adj *p* < 0.05). COL11A1, for instance, was expressed widely throughout the cochlea (Fig. 5G). We conclude from this high expression across many cell types in the inner ear that the MD network + recurrent prioritized genes may play key roles across the ear in Ménière's Disease, and that the disorder may be both polygenic and poly-cellular. However, we acknowledge that this dataset lacks broad representation of all cell types in the human cochlea [38] and further study of a representative single cell atlas is warranted.

## Discussion

The etiology of MD has eluded researchers for over a century. What is known is that in approximately 5% of cases there is a Mendelian pattern of inheritance. The diagnosis of definite MD is based on clinical criteria and requires the observation of an episodic vertigo syndrome associated with low- to medium-frequency sensorineural hearing loss and fluctuating aural symptoms (hearing, tinnitus and/or fullness), most often unilateral, in the affected ear. In this manuscript, we demonstrate the polygenic nature of this disease and posit potential genes and pathways involved. The unilateral nature of the most common form of MD suggests stochastic effects of gene/protein variants likely influenced by environmental factors [39].

Endolymphatic hydrops, the swelling of the scala media compartment of the cochlea, is a well described pathological finding in patients with MD [40]. Literature abounds regarding the possibilities of overproduction or under resorption of endolymph being the primary mechanism for the hydrops seen in temporal bone specimens of affected patients. Both our network and over-representation analysis of recurrently mutated genes identify cell junction assembly as a strongly enriched pathway, leading us to the hypothesis that endolymphatic hydrops may in fact be the result of “leaky” cell–cell and cell-extracellular matrix contacts leading to an influx of ions/fluids from the scala tympani and/or the scala vestibuli into the scala media. This finding suggests that endolymphatic hydrops may have both genetic and environmental factors that lead to alterations of ion and free water flow resulting in changes in endocochlear potential. Initially, this is reversible leading to fluctuating hearing loss. Ultimately, however, this becomes permanent with resulting sensorineural hearing loss and impaired vestibular function. In addition, the maintenance of the endocochlear potential via the stria vascularis, a highly metabolically active tissue, is critically dependent upon energy derived from ATP. Our analysis demonstrates significant enrichment in ATP-related pathways underscoring the likely role of the lateral wall of the scala media in MD.

Prioritizing MD-related mutated genes by inclusion of biological interaction networks to 481 genes points to *MPHOSPH8* associated with transcriptional suppression, *MYO18A* associated with hair cell–cell junction proteins [41], *TRIOBP* associated with human deafness and essential for thickening bundles of F-actin in rootlets, establishing their mature dimensions and for stiffening supporting cells of the auditory sensory epithelium [42], and *OTOGL* associated with vertigo [43] and midfrequency hearing loss [44], as key MD genes. In addition, disruptions in genes instrumental to otic capsule and temporal bone development may lead to sensorineural hearing loss, dizziness, and vertigo; key symptoms of MD [45].

Limitations of this project include lack of healthy control individuals screened negative for MD, which we have attempted to overcome with rigorous statistical analyses leveraging public datasets. However, due to the prevalence of MD in the population (1.2%), individuals with MD or at-risk of developing MD may be included in these public datasets. In addition, our sample population is predicted to be of 96.6% European descent, limiting the broad applicability of these results to other populations, which warrants further study to include individuals from diverse populations. Finally, this analysis is focused on protein altering variants, which likely does not encompass all of the potential molecular mechanisms underlying the physiological changes driving the onset of endolymphatic hydrops.

## Conclusions

Ménière's disease remains an enigma in the field with very little known about the biology and thus, no targeted therapy exists. This study, the first large scale sequencing project on well-characterized unilateral MD, uncovers new genes and pathways underpinning the complex polygenic disease. The genes and pathways we have implicated in MD include known deafness and vertigo genes, genes involved in cell–cell adhesion and the extracellular matrix, stereociliary structure and function, and cellular energy maintenance. Specifically, we hypothesize the disease is driven by abnormally porous cell junctions in the organ of Corti and impaired potassium regulation within the stria vascularis as demonstrated in our tissue specific gene expression data and pathway analysis, and impaired maintenance of the energy stores required for maintaining the necessary tight control of the endocochlear potential. Taken together, this molecular genetic analysis supports several likely mechanisms leading to the final common pathway we see as MD. In the age of precision medicine, these data can be used to create a gene panel for the first objective diagnostic tool for MD. More importantly, these data will allow the scientific community to begin to develop model systems that will lead to targeted therapies.

## Subjects and methods

### Study design & participants

This study was approved by the Institutional Review Board (no. 01–041 and 10–035) of St. Vincent’s Medical Center. Subjects were chosen based on retrospective chart review as having definite MD defined by fluctuating low-frequency hearing loss on serial audiograms, roaring tinnitus exacerbations prior to an attack of vertigo, and a subsequent attack of vertigo lasting less than 24 h. Subjects meeting the inclusion criteria were mailed informed consent forms and saliva collection kits. A total of 1,200 patients provided informed consent and returned saliva collection kits for DNA extraction. 527 of these well characterized and deidentified samples were used for the analysis. Only patients with definite Ménière's disease according to the American Academy of Otolaryngology-Head and Neck Surgery criteria were included.

### Audiometric assessment by evaluation

Standard pure-tone audiometry and word recognition score (WRS) testing (NU-6 25-word lists) via earphones were administered. Audiometric equipment was calibrated yearly, per ISO 1910.95 standards. Pure-tone average (PTA) threshold data were calculated from four frequencies (0.5, 1, 2, and 3 kHz). The number of evaluations ranged from one to 87 separate hearing tests for the sample cases. Individuals with only one evaluation in the data set were not included in the study. A sample of the audiometric distribution was previously published[46].

### Whole genome sequencing

Genomic DNA was acquired from saliva samples submitted and purified according to Pure Gene (Qiagen) standard protocols. All samples were initially purified using Ampure XP beads (0.8:1 sample to bead ratio). Genomic DNA quality was assessed using Genomic DNA Screen Tape on an Agilent 4200 (Agilent Technologies, Santa Clara, CA, USA), and quantity using the Qubit dsDNA HS (High Sensitivity) assay. Samples with DNA Integrity Number (DIN) greater than 4.0 and at least 500 nanogram (ng) of DNA were selected for subsequent processing. 500 ng of Genomic DNA from each sample was fragmented by Adaptive Focused Acoustics (E220 Focused Ultrasonicator, Covaris, Woburn, Massachusetts) to produce an average fragment size of 400 basepairs (bp). Sequencing libraries were generating using the KAPA Hyper Prep Kit (KAPA Biosystems, Wilmington, MA, USA) following manufacturer’s instructions using 3 cycles of amplification. The quality of the library was assessed using High Sensitivity D1000 kit on a 4200 TapeStation instrument. Sequencing was performed using the NovaSeq 6000 Sequencing System (Illumina, San Diego, CA, USA), generating 150 bp paired-end reads to obtain 30X average coverage.

### Variant calling

Single nucleotide variants (SNVs) and small insertions and deletions (IN/DELs) were called using bcbio, which implements GATK 4.1.9 best practices pipeline for joint genotyping on the hg38 reference [47]. A total of 511 out of 527 samples passed quality control checks and were included in the analysis. In order to remove false positives identified in the data following the first variant calling step, we performed additional filtering (supplement).

### Identifying recurrent rare variants

Variants were filtered by gnomad v3.1.2, ExAC, 1000 genomes, to exclude variants common in > 5% of the population. Variants deemed "benign" by Sift or Polyphen were removed, and only protein altering variants were retained. We further filtered to variants of unusually high frequency (observed frequency/gnomad expected frequency > 1.3). Sensitivity analysis was conducted to verify that results were not highly sensitive to choice of threshold (Figure S2). We filtered by observed/expected ratio rather than a variant-level *p*-value computed from allele counts in the study cohort and the gnomad control cohort because the observed/expected filter strongly outperformed the p-value filter in recovery of relevant mammalian ontology (MPO) terms (Figure S3).

### Network analysis

The STRING molecular interaction network (version 11.5) was used, with all edges, in a weighted version of network propagation with weights of edges given by confidence [48]. Genes harboring rare damaging mutations as described above were used as seeds for the network propagation algorithm [22], to score all genes. We compared the calculated propagation score to that of a null ensemble in which mutations in each patient are uniformly random, therefore unrelated to MD. We generated 10^4^ independent samples of 511 patients each from the null ensemble, with numbers of mutations the same as observed in the MD cohort for each patient. Genes were then sorted by *z*-score, where genes with large positive *z* are of high interest.

### Enrichment analysis

Pathway and gene ontology enrichment analysis was conducted on filtered gene sets using the GProfiler tool [49]. Gene sets tested were ‘recurrence’ (4 or more obs/exp > 1.3 frequency genes; *N* = 1098 genes), ‘network’ (network propagation z-score > 3; *N* = 1037 genes), or ‘recurrence + network’ (4 or more obs/exp > 1.3 frequency genes and network propagation z-score > 3; *N* = 481 genes).

### Supplementary Information

Supplementary Material 1.

Supplementary Material 2.

Supplementary Material 3.

Supplementary Material 4.

Supplementary Material 5.

## Data Availability

The code generated during this study is available at https://github.com/ucsd-ccbb/Friedman_WGS and archived source code at the time of publication is available from Zenodo 10.5281/zenodo.11555110. The genomic dataset supporting the current study has not been deposited in a public repository due to genomic data sharing constraints, but are available from the corresponding author upon reasonable request. Individual variants have been deposited in ClinVar (Accession Numbers: SCV005049928 – SCV005050105).
