# Supplementary material for: The genomic landscape of Ménière's disease: a path to endolymphatic hydrops
Source: BMC Genomics. 2024 Jun 28;25:646. doi: 10.1186/s12864-024-10552-3 (PMC11212243; doi:10.1186/s12864-024-10552-3)
Supplement: Supplementary file 5 — Supplementary Material 5. [file 12864_2024_10552_MOESM5_ESM.docx]

Supplementary Appendix

# Supplementary Note:

*Additional filters to variant calls*

In order to remove false positives identified in the data following the first variant calling step, we performed additional filtering. Reads from bam files (hg38) were filtered to include only exon-spanning reads. These exon-spanning reads were aligned with BWA-MEM to the CHM13_T2T complete genome assembly amended by the complete sequence of the 8p23 inversion haplotype[^1^](https://paperpile.com/c/UcIZeT/2Fgty). We removed reads with any soft-clipped bases (3’ and 5’) in the read or its mate. We identified variants with BCFtools mpileup to obtain vcf files with T2T coordinates, which were translated back to hg38 coordinates using liftOver. Only variants observed in both the original hg38 whole genome alignment and in the final T2T exon-only alignments were retained. We plot the distribution of the observed ratio ALT/DP, where ALT is the observed frequency of the alternative allele and DP is the depth of sequencing at that site (Figure S1A). The mode at ALT/DP = 0.5 was expected for heterozygous variants; however, the slight asymmetry at very low values indicated contamination with systematic errors. Since each observation is a Bernoulli trial with probability ½, we calculated a one-sided (to avoid removing any homozygous variants) p-value as the probability of observing at most ALT successes in DP Bernoulli trials, and removed all variants with p<0.05.

*Population stratification*

To estimate genetic ancestry, we used bcftools v1.18 to merge individual .vcfs and plink v1.9 to convert .vcf to plink format. The 1000 Genomes (1kG) phase 3 GRCh38 was used as the reference data set. Plink format files of the 1kG dataset were downloaded from https://www.cog-genomics.org/plink/2.0/resources#1kg_phase3. For quality control, a --maf 0.05 filter was applied. The resulting 1kG reference contained 3202 individuals and 8,774,151 variants. Quality control included --maf 0.05 --geno 0.1 (remove alleles with < 0.05% MAF and remove markers with > 10% missingness). We found the intersection of variants between individuals in our dataset and 1kG reference and matched the build between datasets using plink --update-map. We resolved strand differences between the MD dataset and 1kG reference to generate a list of non-highly-correlated SNPs using plink --indep-pairwise 50 5 0.2 and extracted these from the 1kG reference. We merged 1kG and MD individuals and calculated MDS coordinates using plink commands --cluster --mds-plot 10 while supplying the independent SNP genome previously mentioned to the --read-genome parameter. We then used ADMIXTURE v1.3.0[^2^](https://paperpile.com/c/UcIZeT/zoQQF) to infer ancestry of placenta dataset individuals. Of the 511 samples in the study cohort, 494 were estimated to be of European ancestry (EUR), 20 were of east Asian ancestry (EAS), 14 were estimated to be of American ancestry (AMR), and 3 were estimated to be of African ancestry (AFR). Before filtering for unusually high frequency (>1.3-fold higher than expected), the three samples with African ancestry had more variants per sample than the other ancestries (Figure S4B). After applying the 1.3-fold observed by expected allele frequency filter, the number of variants observed per sample was equivalent across ancestries (Figure S4C).

*Independent validation*

Datasets for external validation of prioritized genes were assembled from a variety of sources. Genes from the otoscope gene panel were downloaded from the NIH (otoscope V9[^3^](https://paperpile.com/c/UcIZeT/83qB6)). Genes associated with MD in the UK Biobank were downloaded from GeneBass[^4^](https://paperpile.com/c/UcIZeT/18LKc) (SKAT p<0.01), where the MD phenotype was self-reported. Gene burden results from LOF variants were tested separately from missense variants. Mouse variant data was downloaded from the Mouse Genome Informatics (MGI) database [^5^](https://paperpile.com/c/UcIZeT/ceEtl), a database connecting genotype to phenotype in mice (using the mammalian phenotype hierarchy). MGI data were parsed using functionality from the NetColoc tool[^6^](https://paperpile.com/c/UcIZeT/NORJz). Genes associated with relevant human phenotypes were downloaded from the GWAS catalog [^7^](https://paperpile.com/c/UcIZeT/Spt9K). Enrichment analysis for the external databases (mouse variant database, otoscope gene panel, UKBB MD genes, GWAS catalog genes) were evaluated with hypergeometric tests.

*External single cell datasets*

The scRNAseq counts data for the human inner ear atlas was downloaded from GEO (GSE213796). These counts data were normalized, integrated, clustered, and UMAP coordinates were computed using Seurat[^8^](https://paperpile.com/c/UcIZeT/HbKND). Cell type IDs were provided by the authors of the original study[^9^](https://paperpile.com/c/UcIZeT/jJ1fr). The scRNAseq differential expression results from the hearing impaired mouse model were provided in supplemental materials of the original manuscript[^10^](https://paperpile.com/c/UcIZeT/4w5MH).

*Multiplexed error-robust fluorescence in situ hybridization (MERFISH) on the cochlear tissue*

MERFISH samples were prepared in accordance with company instructions (Vizgen, Cambridge, MA, USA). Briefly, C57Bl/6J mice cochleae of postnatal day 5 were harvested and fixed with 4% paraformaldehyde (PFA) in 0.1 M phosphate-buffered saline (PBS; pH 7.4) at 4 °C overnight. Samples were dehydrated in graded-sucrose series (10% and 20% for 30 min, and 30% for overnight at 4 °C) with RNase inhibitor [New England Biolabs (NEB), M0314L, Ipswich, MA, USA]. Samples were placed in a cryomold and embedded with O.C.T. compound (Sakura Finetek, Torrance, CA, USA), then frozen with dry ice/ethanol bath. The embedded tissue was sectioned into 10μm thick slices using a Leica CM1860 (Leica Biosystems, Nussloch, Germany) cryostat, and 3 to 5 cochlear mid-modiolar sections were mounted onto a center of MERSCOPE slide glass (Vizgen, #20400001, USA). The mounted sections were washed with 0.1 M PBS, then permeabilized in 70% ethanol at 4 °C for 24 h. The cell boundary staining was performed by using a primary antibody mix (Vizgen, #20300010, USA), followed by a secondary antibody mix (Vizgen, #20300011, USA) for 1 h at 23 ℃, respectively. Stained samples were incubated with an encoding probe [MERSCOPE 140 Gene Panel Mix (Vizgen, #20300006, USA)] for 36 hours at humidified 37 ℃ cell culture incubator. After post-encoding hybridization wash with formamide wash buffer (Vizgen, #20300002, USA), samples were embedded with a gel embedding solution [gel embedding premix (Vizgen, #20300004, USA), 5 ml; 10% ammonium persulfate solution (Millipore-Sigma, 09913-100G, Burlington, MA, USA), 25 µl; N,N,N’,N’-tetramethylethylenediamine (Millipore-Sigma, T7024-25ML, USA), 2.5 µl]. For tissue clearing, samples were incubated in digestion premix (Vizgen, #20300005, USA) with RNase inhibitor (NEB, USA) for 1 h at 23 °C, followed by clearing premix [clearing premix (Vizgen, #20300003, USA), 5ml; proteinase K (NEB, P8107S, USA), 50l] for 48 h at humidified 37 ℃ cell culture incubator. After the tissue became transparent, samples were washed with the wash buffer (Vizgen, #20300001, USA) and incubated with 4′,6-diamidino-2-phenylindole (DAPI) and polythymine (polyT) staining reagent (Vizgen, #20300021, USA) for 15 min with agitation. Images were taken by using MERSCOPE (Vizgen, USA). DAPI / polyT and cell-boundary staining 2 was utilized for the cell segmentation parameter respectively, then image processing analysis was done on the MERSCOPE. The images were visualized and analyzed on the MERSCOPE Visualizer (Vizgen, USA).

# Supplemental figures


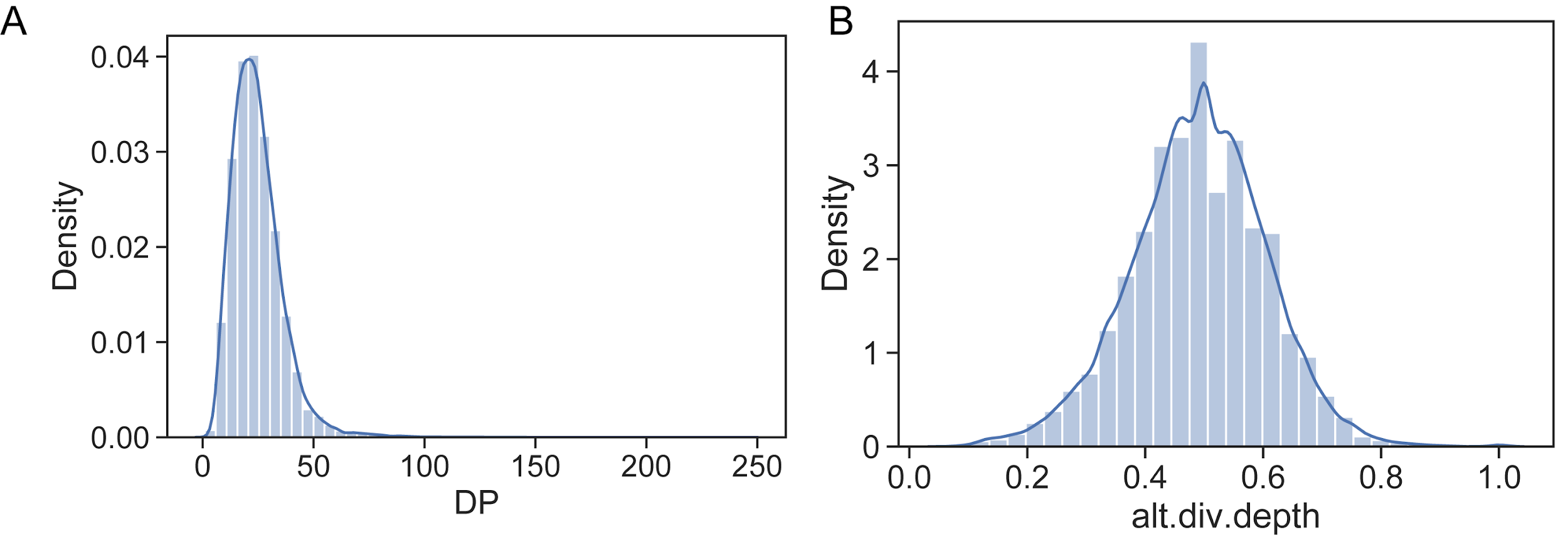


**Figure S1:** A) Distribution of read depth for filtered variants. B) Distribution of number of alternate alleles observed divided by depth, showing the expected normal distribution around 0.5.


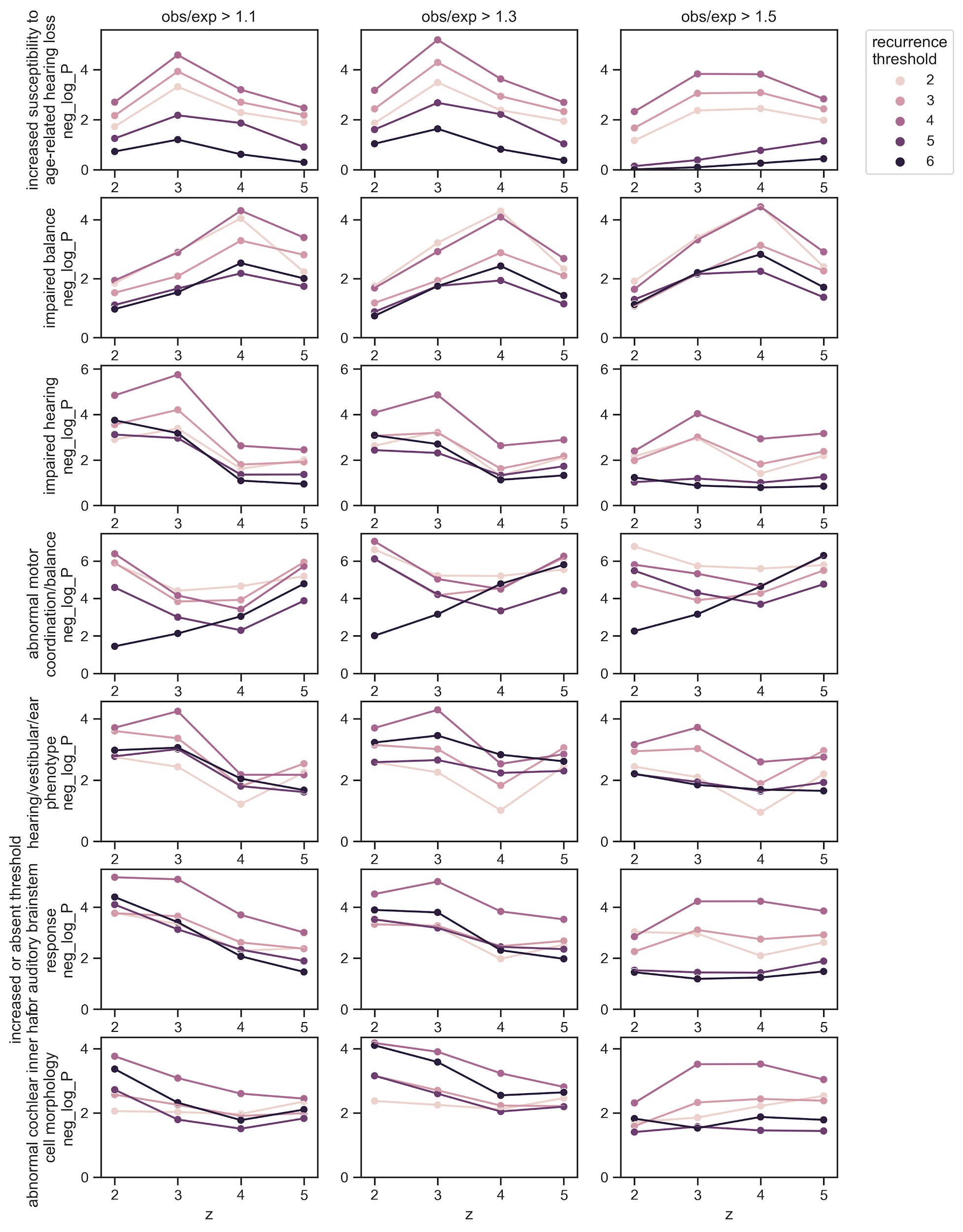


**Figure S2:** Sensitivity analysis to choices of thresholds. Negative log p for enrichment in relevant MPO phenotypes shown for each combination of thresholds.

**
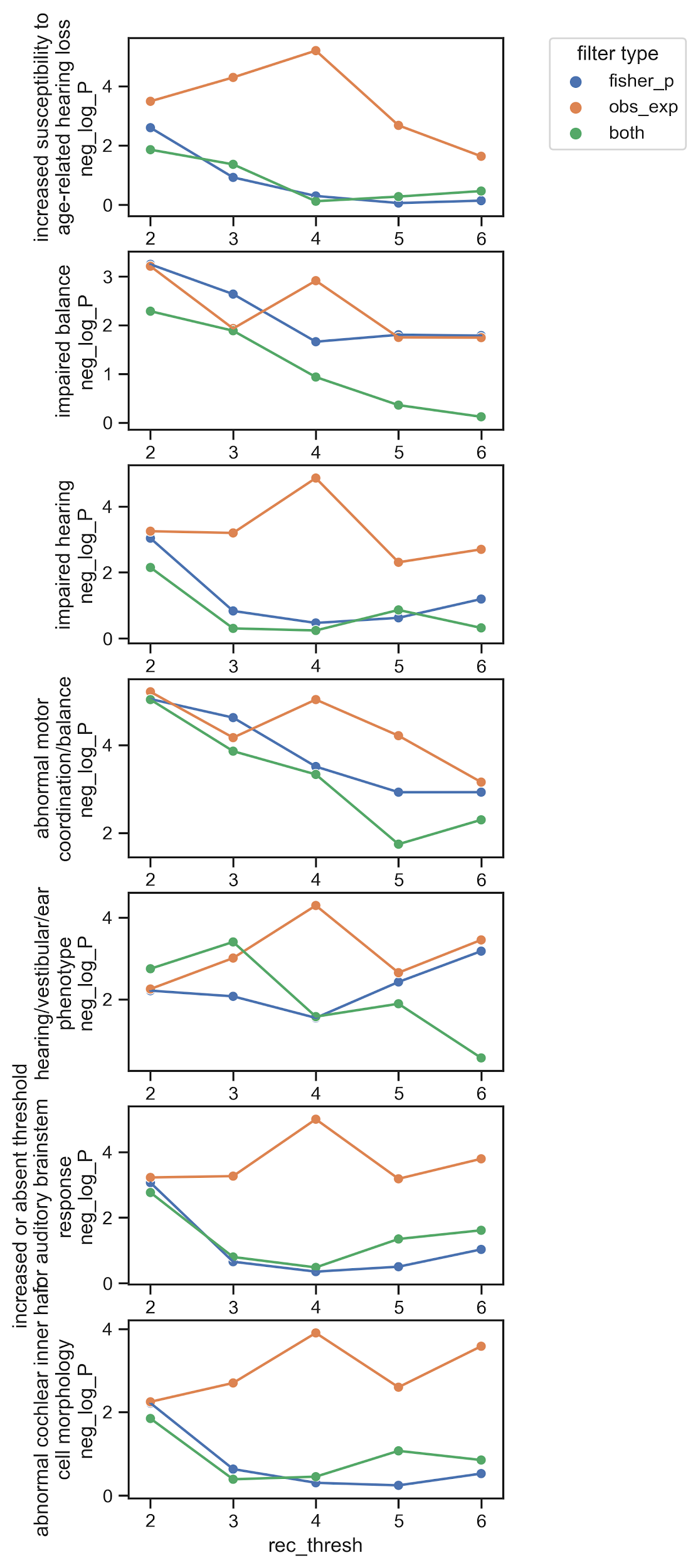
**

**Figure S3:** Comparison of performance of different filtering types in recovery of relevant MPO phenotypes.


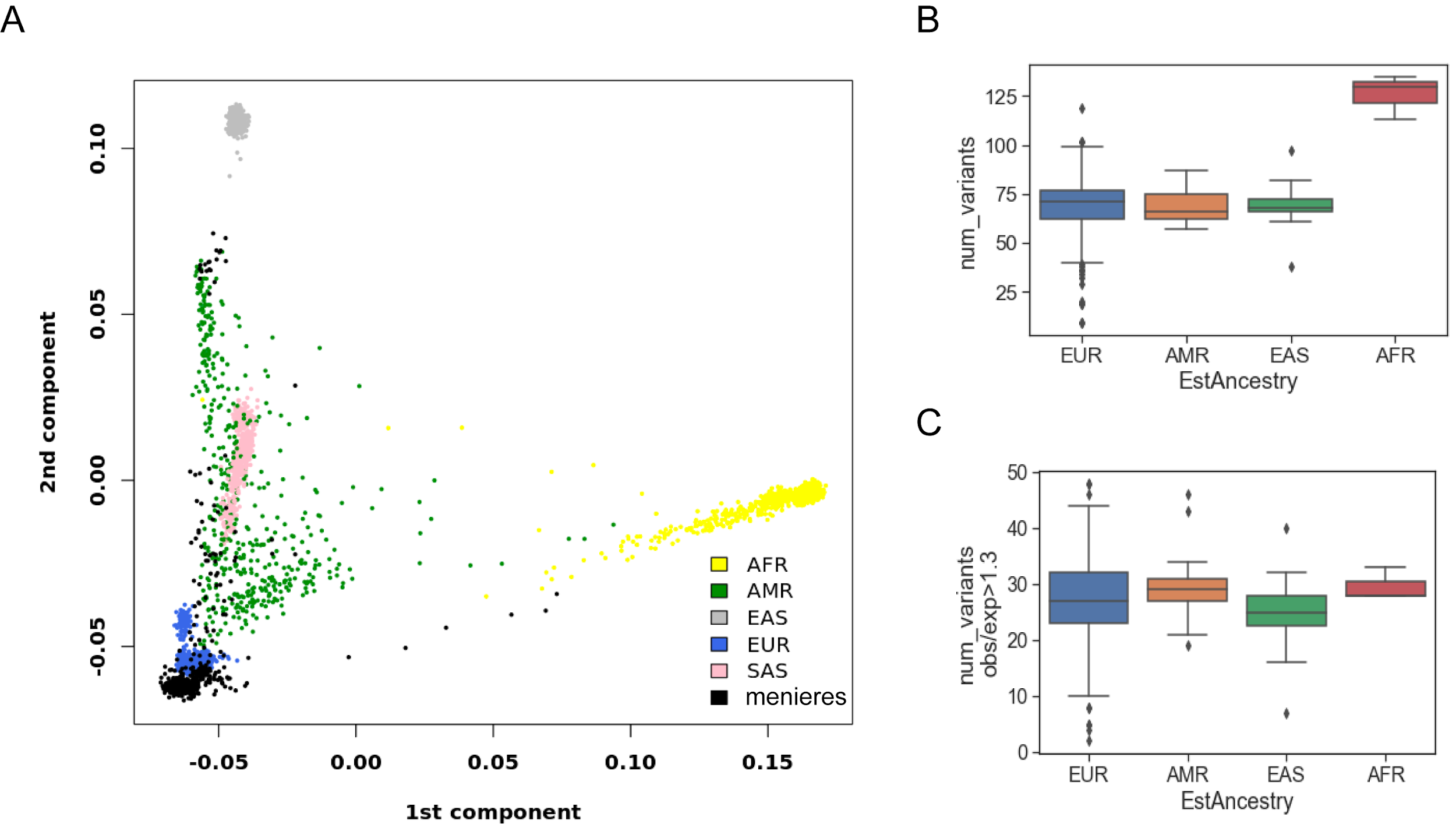


**Figure S4:** Population stratification. A) PCA plot showing the projection of the 511 Menieres samples onto the coordinates defined by 1000 genomes. B) Boxplots showing the number of variants per ancestry, before filtering. C) Boxplots showing the number of variants per ancestry, after filtering.


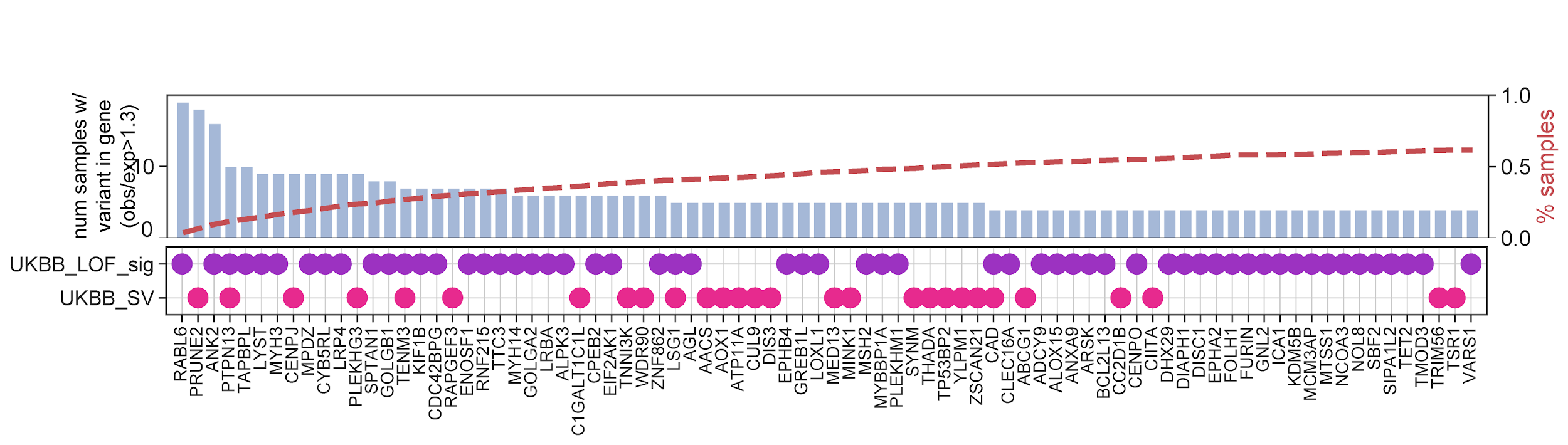


**Figure S5:** Genes in the recurrent set (4 or more variants with obs/exp frequency > 1.3) and in either the UKBB MD LOF gene burden significant set (dark purple), or the UKBB MD single variant set (pink). Barchart shows the number of samples with a variant in focal gene (left axis), with the cumulative sum (% of all samples) on the right axis.

#

# References

1. [Mohajeri K, Cantsilieris S, Huddleston J, et al. Interchromosomal core duplicons drive both evolutionary instability and disease susceptibility of the Chromosome 8p23.1 region. Genome Res 2016;26(11):1453–67.](http://paperpile.com/b/UcIZeT/2Fgty)

2. [Alexander DH, Lange K. Enhancements to the ADMIXTURE algorithm for individual ancestry estimation. BMC Bioinformatics 2011;12:246.](http://paperpile.com/b/UcIZeT/zoQQF)

3. [OtoSCOPE v9 [Internet]. [cited 2023 Oct 19];Available from:](http://paperpile.com/b/UcIZeT/83qB6) <https://www.ncbi.nlm.nih.gov/gtr/tests/593050/>

4. [Karczewski KJ, Solomonson M, Chao KR, et al. Systematic single-variant and gene-based association testing of thousands of phenotypes in 394,841 UK Biobank exomes. Cell Genom 2022;2(9):100168.](http://paperpile.com/b/UcIZeT/18LKc)

5. [Eppig JT, Smith CL, Blake JA, et al. Mouse Genome Informatics (MGI): Resources for Mining Mouse Genetic, Genomic, and Biological Data in Support of Primary and Translational Research. Methods Mol Biol 2017;1488:47–73.](http://paperpile.com/b/UcIZeT/ceEtl)

6. [Rosenthal SB, Wright SN, Liu S, et al. Mapping the common gene networks that underlie related diseases. Nat Protoc [Internet] 2023;Available from:](http://paperpile.com/b/UcIZeT/NORJz) <http://dx.doi.org/10.1038/s41596-022-00797-1>

7. [Welter D, MacArthur J, Morales J, et al. The NHGRI GWAS Catalog, a curated resource of SNP-trait associations. Nucleic Acids Res 2014;42(Database issue):D1001–6.](http://paperpile.com/b/UcIZeT/Spt9K)

8. [Satija R, Farrell JA, Gennert D, Schier AF, Regev A. Spatial reconstruction of single-cell gene expression data. Nat Biotechnol 2015;33(5):495–502.](http://paperpile.com/b/UcIZeT/HbKND)

9. [van der Valk WH, van Beelen ESA, Steinhart MR, et al. A single-cell level comparison of human inner ear organoids with the human cochlea and vestibular organs. Cell Rep 2023;42(6):112623.](http://paperpile.com/b/UcIZeT/jJ1fr)

10. [Sun G, Zheng Y, Fu X, et al. Single-cell transcriptomic atlas of mouse cochlear aging. Protein Cell 2023;14(3):180–201.](http://paperpile.com/b/UcIZeT/4w5MH)
